# In-silico design of an immunoinformatics based multi-epitope vaccine against *Leishmania donovani*

**DOI:** 10.1186/s12859-022-04816-6

**Published:** 2022-08-05

**Authors:** Subhadip Saha, Shubham Vashishtha, Bishwajit Kundu, Monidipa Ghosh

**Affiliations:** 1 Department of Biotechnology, National Institute of Technology Durgapur, Durgapur, 713209 India; 2 Kusuma School of Biological Sciences, Indian Institute of Technology Delhi, Hauz Khas, New Delhi, 110016 India

**Keywords:** Visceral leishmaniasis, *Leishmania donovani*, Immunoinformatics, Vaccine, Molecular docking

## Abstract

**Background:**

Visceral Leishmaniasis (VL) is a fatal vector-borne parasitic disorder occurring mainly in tropical and subtropical regions. VL falls under the category of neglected tropical diseases with growing drug resistance and lacking a licensed vaccine. Conventional vaccine synthesis techniques are often very laborious and challenging. With the advancement of bioinformatics and its application in immunology, it is now more convenient to design multi-epitope vaccines comprising predicted immuno-dominant epitopes of multiple antigenic proteins. We have chosen four antigenic proteins of *Leishmania donovani* and identified their T-cell and B-cell epitopes, utilizing those for in-silico chimeric vaccine designing. The various physicochemical characteristics of the vaccine have been explored and the tertiary structure of the chimeric construct is predicted to perform docking studies and molecular dynamics simulations.

**Results:**

The vaccine construct is generated by joining the epitopes with specific linkers. The predicted tertiary structure of the vaccine has been found to be valid and docking studies reveal the construct shows a high affinity towards the TLR-4 receptor. Population coverage analysis shows the vaccine can be effective on the majority of the world population. *In-silico* immune simulation studies confirms the vaccine to raise a pro-inflammatory response with the proliferation of activated T and B cells. *In-silico* codon optimization and cloning of the vaccine nucleic acid sequence have also been achieved in the pET28a vector.

**Conclusion:**

The above bioinformatics data support that the construct may act as a potential vaccine. Further wet lab synthesis of the vaccine and in vivo works has to be undertaken in animal model to confirm vaccine potency.

**Supplementary Information:**

The online version contains supplementary material available at 10.1186/s12859-022-04816-6.

## Background

Leishmaniasis is a vector-borne chronic inflammatory disease, caused by the protozoan parasite of the *Leishmania* genus, transmitted to humans by sandflies [1]. The genus *Leishmania* belongs to a family of Trypanosomatidae of the order kinetoplastida [2]. Sandflies mainly of the genus, *Phlebotomus* and *Lutzomyia are* known to spread this disease [3]. Visceral Leishmaniasis (VL) is the deadliest form of leishmaniasis which is caused by *Leishmania donovani* in South Asia (India, Bangladesh, Nepal) and East Africa (Sudan, Ethiopia, Somalia, and Kenya) and by *Leishmania infantum* in the Middle East, Mediterranean, Brazil, Pakistan and Iran [4]. The disease is endemic in more than 60 countries, with an estimated 50,000–90,000 new cases of visceral leishmaniasis occurring each year worldwide [5, 6]. 90% of worldwide visceral leishmaniasis cases have been reported in Ethiopia, India, Bangladesh, South Sudan, and Sudan, Brazil, Kenya [7]. In these countries, leishmaniasis is a significant health issue and sets a huge impact on socio-economic conditions. More than 50,000 annual deaths due to leishmaniasis have been reported by The Global Burden of Disease Study (GBD) in 2010 [8]. Currently, anti-leishmanial therapies, based on chemotherapeutics are available but drug toxicity and numerous side effects set a huge drawback [9]. Pentavalent Antimonials which are widely used drugs for VL is being proven less effective due to drug resistance mainly in India [10]. Application of Amphotericin B provides a cure rate of more than 98 percent but causes fever, chills, thrombophlebitis, and in serious cases nephrotoxicity [11]. Another widely used and successful VL drug Miltefosine tends to have teratogenic effects in animals [12].

A licensed vaccine for leishmaniasis is a special need for society to successfully eradicate the disease. Currently, there is no licensed vaccine for leishmaniasis in humans [13]. Several vaccine development strategies including live attenuated vaccine, DNA vaccine, and proteins have been tried and tested in animal models [14–16]. Recombinant protein vaccine based upon Leishmania analogue of the receptor kinase C (LACK), Thiol-specific-antioxidant (TSA), Glycoprotein 63 (GP63), GP46, M2, *L. major* homologue of eukaryotic stress-inducible protein-1(LmST1), and Leish111f has been evaluated as an effective vaccine candidate to offer protective immunity against leishmaniasis [17, 18]. Leish111f is a polyprotein vaccine composed of LeIF, TSA, and LmSTI1. Several other VL antigens like Cysteine Protease C, Stage-Specific S antigen, and Kinetoplastid membrane protein have got limited success in terms of vaccine potency [19]. Leishmune® and CaniLeish® are two fractionated first-generation licensed vaccines that induce a protective immune response in dogs and can block the transmission of the parasite from dogs to humans [1, 20].

Immunoinformatics based computational vaccinology involves mapping immuno-dominant B and T cell epitopes in an antigenic protein, analyzing their immunogenic potential, merging them with appropriate linkers, and measuring their ability to bind host cell receptors, indicating that it can be a new tool for vaccine development. Immunoinformatics utilizes certain epitopes of proteins to construct a chimera instead of using whole proteins/antigens. Administration of these vaccines can later elicit efficient T-cell and B-cell mediated secondary responses if contacted with specific pathogen derived antigens. Adu-Bobie et al., 2003 [21] published one of the first reports on a successful immuno-bioinformatics-based vaccine against *Neisseria meningitides.* Solanki et al., 2018 [22] used subtractive proteomics to build a chimeric vaccine by integrating promiscuous membrane antigens against *Acinetobacter baumanii.* Recently a promising immunoinformatics-based multi-epitope malaria vaccine incorporating 1 B-cell epitope, 12 CD4^+^ T-Helper cell epitopes, and 10 CD8^+^ cytotoxic T-cell epitopes has been constructed by Maharaj et al., 2021 [23]. Mahapatra et al., 2020 [24] designed a multi-epitope vaccine targeting SARS-COV2 showing high population coverage by mapping potential B and T cell epitopes of Spike, membrane, nucleocapsid, and envelope protein of the virus. For VL several in-silico study-based vaccines have been reported. A multi-epitope vaccine consisting of immunogenic epitopes obtained from secretory proteins of *L. donovani* has been reported by khatoon et al., 2017 [9] and khan et al., 2020 [25] with their probable immunogenic and antigenic properties. A multi-epitope subunit vaccine consisting of *L. donovani* hypothetical proteins has been analyzed previously and has been found to be immunogenic and non-allergic with probable potential to raise both humoral and cell-mediated immunity [26]. Brito et al., 2020 [27] used immunoinformatics to design two chimeras consisting of epitopes of acid ribosomal protein P2, acid ribosomal protein P0, Leishmania homologue of activated C kinase, cysteine peptidase C, and histone H2A as chimera A, and cysteine peptidase proteins A and B, surface antigenic protein, and specific amastigote protein A2 as chimera B and showed in *in-vivo* models that the chimeras can induce T cell proliferation and cytokine expression as well as facilitate parasite clearance.

In this paper, we evaluated the potential of an immunoinformatics-based chimeric vaccine integrating immuno-dominant epitopes of four *L. donovani* proteins, an ATP-dependent Zinc Metallopeptidase, and a Histidine Secretory Acid Phosphatase, a Rhomboid like protein, and an Amastin-like surface protein (ALSP). Prior to this we went through previous literature and found a surface zinc metalloprotease called GP63 or Leishmanolysin which facilitates *Leishmania* survival inside macrophages, helps in complement-mediated lysis evasion, and confers resistance against antimicrobial peptides [28–30]. The particular sequences of the proteins were selected owing to the predicted antigenicity during a pre-screening analysis. It has been reported in several papers that GP63 zinc metalloprotease is a potent vaccine candidate against *Leishmania* [31–33]. These observations open the door for the evaluation of other *L. donovani* zinc metalloproteases as vaccine targets. Besides this Rhomboid protease belonging to serine proteases has been studied before in *Toxoplasma gondii* and its significant role in facilitating parasite entry into the cell has been indicated [34]. DNA vaccine with Rhomboid antigen against *T. gondii* has been found to successfully raise humoral and cellular response with enhanced pro-inflammatory cytokine expression [35, 36]. The ALSP has been purified previously and found to be a lipase (Pre-print). It is expressed specifically in the amastigote stage indicating its probable role in visceralisation [37]. Acid ecto-phosphatases, a member of Histidine Acid Phosphatase have been reported to confer resistance to oxidative stress by inhibiting Neutrophil superoxide anion production [38]. Singla et al., 1992 [39] observed a correlation between Histidine acid phosphatase and virulence of *L. donovani,* indicating membrane-bound acid phosphatase activity of virulent promastigotes are higher than the avirulent one. Recently it has been found that *L. donovani* membrane-bound acid phosphatase overexpression facilitates parasite survival in macrophages [40].

We obtained the fasta files of the four proteins from the NCBI database and selected possible antigenic Helper T-lymphocytes (HTL) and Cytotoxic T-lymphocytes (CTL) epitopes of the proteins using various web servers like Immune Epitope Database (IEDB), NETMHCII 2.3, NETMHC 4.0, and NETCTL based on various factors which are described later on. Apart from this we also tried to figure out possible B-cell epitopes for potent antibody response. The selected epitopes were fused with suitable linkers and a toll-like receptor-4 (TLR-4) agonist 50S ribosomal protein L7/L12 was attached to the N-terminal region to form a chimeric vaccine. After several physicochemical characterizations, the tertiary structure of the vaccine is modeled, refined, and docked with TLR-4 receptor for analyzing the binding affinity of the vaccine with the receptor. Lastly, Molecular Dynamics (MD) simulation was carried out to evaluate the stability of the docked complex in-silico.

## Results

### Antigenicity analysis of selected proteins

Antigenicity is one of the key parameters which is applied while considering a protein for vaccine designing. The sequences of all the four proteins were uploaded to Vaxijen server and the default threshold of 0.5 for parasites was maintained. Table 1 implies the predicted antigenicity of each protein.

**Table 1:** Predicted Antigenicity of selected proteins

Sl. No	Protein ID	Vaxijen antigenicity score
1	XP_003863163	0.57
2	XP_003865788	0.60
3	XP_003857936	0.55
4	XP_003864424	0.78

### HTL and CTL epitope prediction

A variable number of Major histocompatibility complex (MHC) class II binding epitopes were obtained for each of four proteins having percentile rank ≤ 1 and IC50 value of ≤ 50 from the Immune epitope database (IEDB). Among them, a total of 93 epitopes from all four proteins were found to be antigenic and hence shortlisted. Among the filtered antigenic epitopes, 35 were found to be IFN-γ inducing and IL-10 non-inducing. These 35 epitopes were applied to the NETMHCII server and again screened for their binding affinity. 6 epitopes were chosen for the construction of the final vaccine based on their ability to bind a wider number of alleles which were included in the study as predicted by the NETMHCII server. The selected epitopes are displayed in Table 2. Similarly, for CTL epitopes the protein sequences were uploaded to the NETCTL server and screened against allelic subtypes A1, A2, A3, and B7. A total of 49 epitopes for A1, 64 epitopes for A2, 61 epitopes for A3, and 60 epitopes for B7, having a threshold value above 0.75, were identified for the four proteins. The identified epitopes were screened for antigenicity and immunogenicity using Vaxijen and IEDB class I immunogenicity server. In ATP-dependent Zinc Metallopeptidase, Histidine Secretory Acid Phosphatase, and Rhomboid-like protein, we obtained 12 epitopes for A1, 24 epitopes for A2, 17 epitopes for A3, and 12 epitopes of B7 which were immunogenic and antigenic. In Amastin-like surface protein majority of the epitopes failed to qualify the criterion of combined immunogenicity and antigenicity, as a result of which only one B7 subtype epitope “SPWLSPIPH” which was predicted to be highly antigenic but not enough immunogenic was taken into consideration for further analysis. The selected epitopes were again analyzed in the NETMHC 4.0 server and their binding affinity was noted against a wider section of alleles. From NETMHC 4.0 server epitopes were carefully chosen in a way to maximize the number of alleles that will have binding efficacy against the CTL region of the vaccine similar to what was done for the HTL region. Table 3 sums up the potential CTL epitopes considered.

**Table 2:** HTL Epitopes shortlisted for Vaccine construction

Sl. No	Protein ID	Selected Epitopes	IEDB Percentile Rank	SMM Align IC_50_	Vaxijen Score	Binding Alleles^#^	IFN- Pred Score	IL-10 Induction
1	XP_003863163	EIKEAVLYENYLQVY	0.32	18	1.037	HLA-DPA1*0201-DPB1*0101, HLA-DPA1*0103-DPB1*0201, HLA-DPA1*0103-DPB1*0401, HLA-DPA1*0301-DPB1*0402, HLA-DPA1*0201-DPB1*0501 DRB1*1501, DRB1*1201, DRB1*1302, HLA-DQA1*0501-DQB1*0201, HLA-DQA1*0101-DQB1*0501	0.108	X
2	XP_003865788	MRPPFAVTIFVELLQ	0.08	10	1.312	HLA-DPA1*0201-DPB1*0101, HLA-DPA1*0103-DPB1*0401, HLA-DQA1*0401-DQB1*0402, HLA-DQA1*0301-DQB1*0302, HLA-DPA1*0103-DPB1*0201, HLA-DPA1*0301-DPB1*0402, HLA-DPA1*0201-DPB1*1401.	0.180	X
		YVRLIRGNPVKTANG	0.70	49	1.039	DRB1*0101, DRB1*1302, DRB1*1501, DRB1*0401 DRB1*0701, DRB1*0802, DRB1*0901, DRB1*1101, DRB1*1201, DRB3*0202, DRB4*0101, HLA-DQA1*0501-DQB1*0301, HLA-DQA1*0102-DQB1*0602	0.361	X
3	XP_003857936	CRRLRLAQSRRRAAQ	0.02	4	0.918	DRB1*0301, DRB1*1101, DRB4*0101, DRB5*0101, DRB1*0101, DRB1*0405, DRB1*0901, DRB3*0202	0.386	X
		LFVWAVGGAAGGLCC	0.31	8	0.825	HLA-DQA1*0501-DQB1*0301, DRB1*0101, DRB1*0901	0.479	X
4	XP_003864424	ASSPFSSTRSSSSSR	0.93	17	0.762	DRB1*0405, DRB1*0401, DRB1*0701.	0.626	X

^#^Strong Binding alleles are labeled in red and weak binders in black

**Table 3:** CTL Epitopes shortlisted for Vaccine construction

Sl No	Protein ID	Allele supertype	Epitopes	Vaxijen score	Binding alleles ^#^	IEDB Immunogenicity Score
1	XP_003863163	A1	MTTIRCLSY	0.503	HLA*A0101,HLA*B5801, HLA*B3501, HLA*B1501	0.040
		A2	IMIAITVVL	0.842	HLA-A0201, HLA*A0202, HLA*A0211, HLA*A0212, HLA*B3901, HLA*B1501 HLA*A0203, HLA*A0206, HLA*A0216, HLA*A0217, HLA*A0219, HLA*B5801 HLA*B3501	0.307
		A3	MSYAVEATK	0.592	HLA*A0301, HLA*B5801	0.228
		B7	LPMAADTPA	0.635	HLA*B0702, HLA*B3501, HLA*B3503	0.067
2	XP_003865788	A2	FQDDYFYPV	1.756	HLA*A0201 HLA*A0202 HLA*A0203 HLA*A0205 HLA*A0206 HLA*A0207 HLA*A0211 HLA*A0212 HLA*A0216 HLA*A0217 HLA*A0219 HLA*A0250 HLA*B2720 HLA*B3901, HLA*A0101, HLA*B3503	0.126
		A3	RLIRGNPVK	1.453	HLA*A0301	0.136
		B7	RPPFAVTIF	1.475	HLA*B0702, HLA*B5802, HLA*B3501, HLA*B3503	0.301
3	XP_003857936	A1	FSAAYYGRF	1.964	HLA*A0101,HLA*B3503, HLA*B3501,HLA*B5801, HLA*B1501	0.103
		A2	RVMRYGFSV	0.595	HLA*A0201, HLA*B2720, HLA*A0206, HLA*A0205, HLA*B0702, HLA*A0250, HLA*A0219, HLA*A0217, HLA*A0216, HLA*A0212, HLA*A0211, HLA*A0207, HLA*A0203, HLA*A0202, HLA*B5801	0.025
		A3	MLYNNFTLA	0.694	HLA*A0201, HLA*A0202, HLA*A0203, HLA*A0206, HLA*A0250, HLA*A0211, HLA*A0212, HLA*A0216, HLA*A0219, HLA*A0301	0.122
4	XP_003864424	B7	SPWLSPIPH	2.064	HLA*B3501, HLA*B0702	-0.004

^#^ Strong binding alleles are labeled in red and weak binders in black

### B-Cell epitope prediction

4 B-cell epitopes were selected of 18-mer length based on score and probable antigenicity obtained from ABCpred and Vaxijen server. The selected B-cell epitopes are given in Table 4.

**Table 4:** Shortlisted B-Cell Epitopes for vaccine construction

Sl No	Protein ID	*B*-cell epitopes	ABCPred score	Vaxijen antigenicity score
1	XP_003863163	WIVPRTCLFVLIMIAITV	0.93	0.876
2	XP_003865788	AGRLDNATNLKAVYPGLM	0.89	0.995
3	XP_003857936	GATDYAAEAERRALRSTW	0.87	0.720
4	XP_003864424	SRLPCGSLCCAPPLHLSQ	0.79	1.176

### Evaluation of epitope conservancy and population coverage analysis

The selected epitopes of all the proteins except ALSP were fairly conserved among four selected strains of *L. donovani*. A moderate level of epitope conservancy was noted in the JPCM5 strain of *L. infantum* and poor conservancy was noted among *L. braziliensis* and *L. major*. ALSP was present only in strain BPK282A1 and absent in other species and strains. Table 5 shows the percentage of epitope sequence homology across the selected leishmania species and strains. HLA allele distribution varies across the world among various ethnic populations and geographical locations thus suggesting the thorough population coverage analysis of epitope vaccine to be crucial. The cumulative coverage of MHC class II binding epitopes was found to be 99.99% and that of class I epitopes was 79.98%. The combined coverage of both T-cells (Both MHC I and II) binding epitopes was 100% against the world population (Additional file 1: Fig. S1).

**Table 5:** Conservancy Analysis of Shortlisted Epitopes

Protein IDs	Selected epitopes	% Sequence homology						
		*L. donovani*				*L. infantum*	*L. major*	*L*. *braziliensis*
		BPK282A1	CL-SL	HU3		JPCM5	FRIEDLIN	*MHOM/BR/75/M2903*
XP_003863163	EIKEAVLYENYLQVY	100	100	100		100	100	86
	MTTIRCLSY	100	100	100		33.3	77	33
	IMIAITVVL	100	100	100		100	66	77.7
	MSYAVEATK	100	100	100		100	100	100
	LPMAADTPA	100	100	100		100	88	55.56
	WIVPRTCLFVLIMIAITV	100	100	100		100	83	83.3
XP_003865788	MRPPFAVTIFVELLQ	100	100	100		100	80	93.3
	YVRLIRGNPVKTANG	100	100	93.3		93.3	80	93.3
	FQDDYFYPV	100	100	100		100	100	77.78
	RLIRGNPVK	100	100	100		100	88.8	100
	RPPFAVTIF	100	100	100		100	77.7	88.8
	AGRLDNATNLKAVYPGLM	100	100	100		100	38.8	61
XP_003857936	CRRLRLAQSRRRAAQ	100	100	100		100	93	53.3
	LFVWAVGGAAGGLCC	100	100	100		93.3	80	73.3
	FSAAYYGRF	100	100	100		100	88.8	66.6
	RVMRYGFSV	100	100	100		33.33	33	44.4
	MLYNNFTLA	100	100	100		100	77.7	77.78
	GATDYAAEAERRALRSTW	100	100	100		100	94.4	61.11
XP_003864424	ASSPFSSTRSSSSSR	100	33.3	33.3		20	NA	NA
	SPWLSPIPH	100	44.4	44.4		22	NA	NA
	SRLPCGSLCCAPPLHLSQ	100	33.3	33.3		NA	NA	NA

### Vaccine construction and secondary structure prediction

**Fig. 1:**
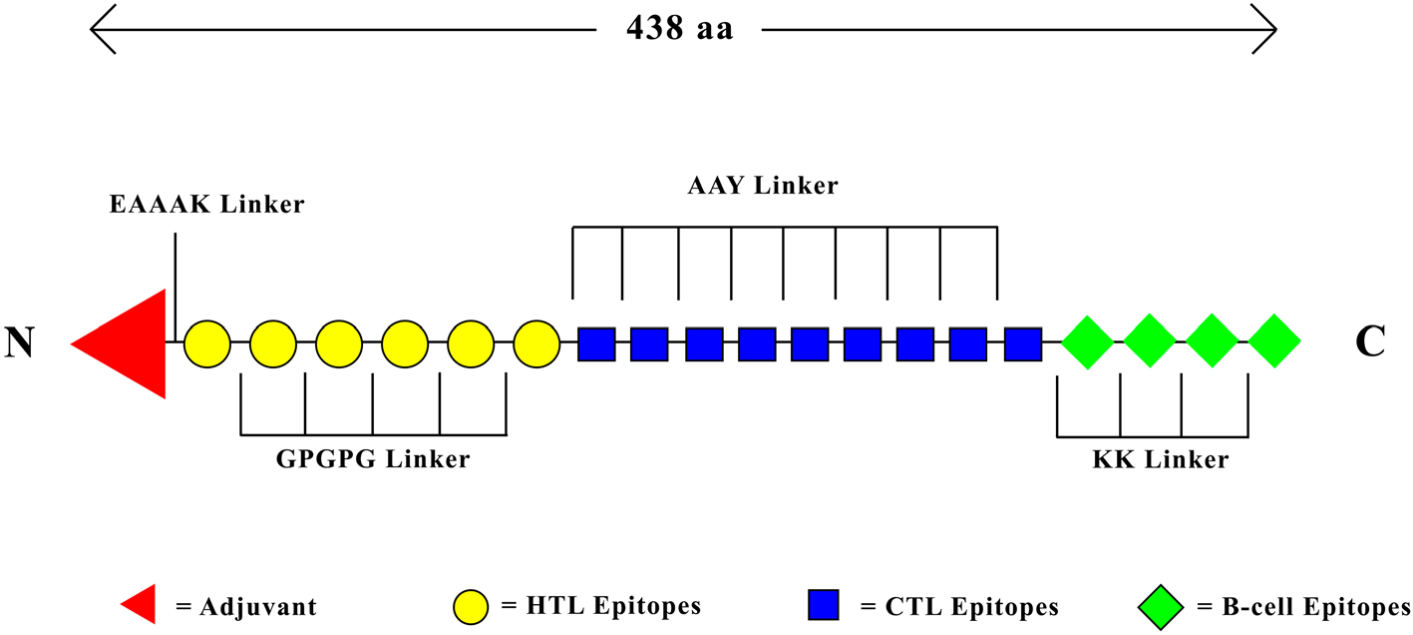
Schematic diagram of Multi-epitope vaccine. 6 HTL epitopes (shown in yellow), 9 CTL epitopes (shown in blue) and 4 B-cell epitopes (shown in green) are joined with GPGPG, AAY and KK linker. Ribosomal L7/L12 adjuvant (Shown in red) is attached to N-terminal end with EAAAK linker

The 438 amino-acid vaccine was constructed using 6 HTL epitopes, 9 CTL epitopes, and 4 B-cell epitopes which met all filtering criteria. Two CTL epitopes of Histidine Secretory Acid Phosphatase, “RLIRGNPVK” and “RPPFAVTIF” which were shortlisted for vaccine construction were found to have already been present in the shortlisted HTL epitope “YVRLIRGNPVKTANG” and “MRPPFAVTIFVELLQ” of the same protein. As a result of this, the two CTL epitopes were removed during the final vaccine construction to avoid redundancy. The HTL epitopes were joined by GPGPG linker, the CTL epitopes with AAY linker, and the B-cell epitopes with KK linker. The AAY linker was also used to join the HTL region to the CTL region, and the KK was used to join the CTL region to the B-cell epitope. Adjuvant 50S ribosomal protein L7/L12 was attached to the N-terminal of the construct by the EAAAK linker (Fig. 1). The protein sequence was uploaded to PSIPRED to predict secondary structure. 54% of alpha-helix, 6% beta-sheets, and 40% coils were obtained as a part of secondary structure prediction from the webserver.

### Physicochemical characterization of vaccine construct and glycosylation pattern analysis

The vaccine construct was to be a probable non-allergen from the AllerTop web server. The other physicochemical properties like Theoretical pI, Instability Index, Molecular Weight, Aliphatic Index, and Grand Average of Hydropathicity (GRAVY) were analyzed using Protparam. The molecular weight of the vaccine was found to be 46 KDa. The theoretical pI was 9.16 which states the vaccine is basic in nature. The instability index of 39.50 suggests that the vaccine is stable. The aliphatic index of the chimeric construct was found to be 87.9 indicating the vaccine to be thermally stable. For evaluation of protein hydrophobicity, the GRAVY score was used. The GRAVY score was found to be 0.121 showing a hydrophobic nature of the protein. Besides this, the in vivo half-life in yeast was > 20 h and in vitro half-life in human reticulocytes was 30 h. Analysis using the NetNglyc indicated that 3 of the selected epitopes “YVRLIRGNPVKTANG”, “AGRLDNATNLKAVYPGLM”, and “MLYNNFTLA” lie within the glycosylated site of their respective target protein sequences.

### Tertiary structure prediction of vaccine and validation

**Fig. 2:**
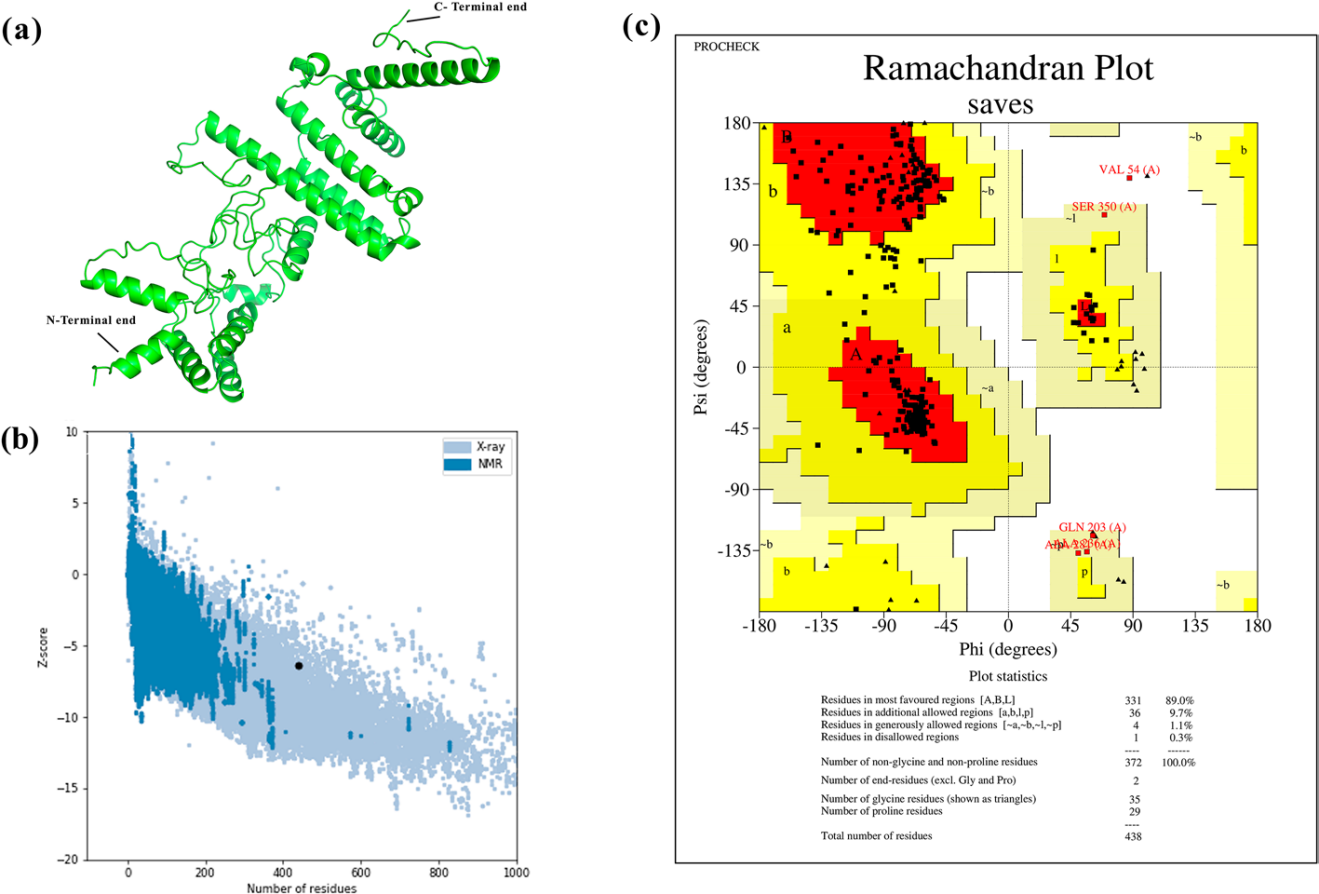
Vaccine 3D model construction and validation. **a** 3D model of multi-epitope vaccine generated by RaptorX server. **b** Z-plot obtained for the refined vaccine model from Pro-SA webserver. Y-axis shows the Z-score obtained by NMR or X-Ray crystallography for native proteins and X-axis is the number of residues. The black dot in the Z-plot denotes the obtained Z- Score of our vaccine **c** Ramachandran Plot of the refined Vaccine 3D model, showing the number of residues in most favourable, Additional favoured, generously favoured and disallowed regions

**Fig. 3:**
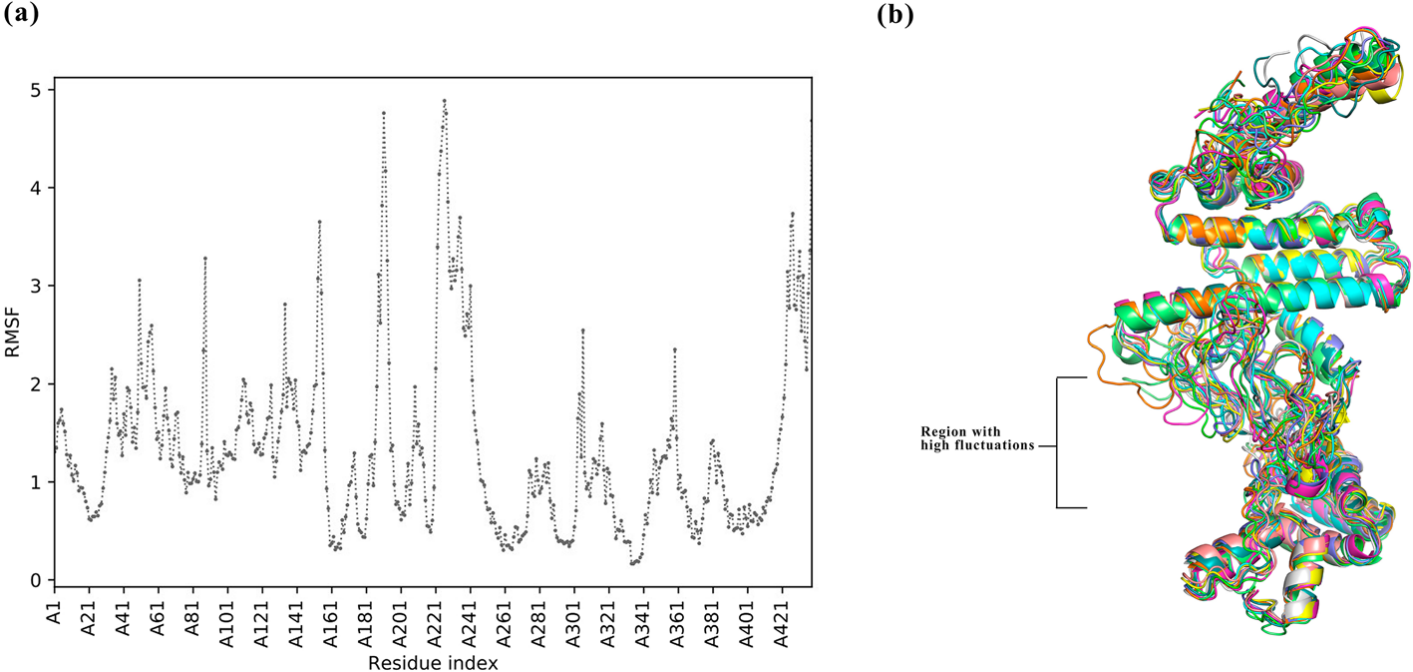
Flexibility simulation of Vaccine model. **a** Root Mean Square Fluctuation (RMSF) plot of the vaccine model obtained after flexibility simulation, denoting the regions with high fluctuations in the construct **b** Superimposition of 10 simulation models obtained from CABS-Flex showing fluctuations through-out

RaptorX server was used to predict the 3D model of the vaccine (Fig. 2a). The Protein Data Bank (PDB) files of the models were downloaded from the server and the output files were validated in ERRAT, PROCHECK, and ProSA tools. The RaptorX model with rank 1, scoring 82 in ERRAT, and produced Ramachandran Plot displaying 83.1% in the favored zone, 13.4% in the additional favored zone, 3% in the generously favored zone and, 0.5% in the disallowed zone was adopted for next studies. The ProSA-web server generated a Z-Score of -6.08 which is in the range of characteristics for native proteins. GalaxyRefine was used to further refine the tertiary model obtained from RaptorX and output model 4 was selected having a Global Distance Test—High Accuracy (GDT-HA) value of 0.9515. The root mean square deviation (RMSD) of the same was found to be 0.434 where a lower value signifies better stability. The MolProbity value of model 4 was 1.937 in contrast to 2.17 of the initial model stating the minimization of error in the 3D model. The refined model produced a Z-score of -6.37 which is still in the range of native proteins (Fig. 2b). The refined model 4 was exported to SAVES version 6 and an ERRAT overall quality score of 89 was obtained with Ramachandran Plot showing 89% in the most favored zone, 9.7% in the additional allowed region, 1.1% in the generously allowed region, and 0.3% in the disallowed region (Fig. 2c). CABS-Flex 2.0 was utilized for fast simulation for flexibility of refined vaccine model. A high amount of fluctuations was noted in the amino acid residues ranging between 133–241 with Leu142, Pro154, Gly173, Ala188, Gly211, Ala225, Gly233, and Gly235 reaching Root mean square fluctuation (RMSF) of 3.44 Å, 3.04 Å, 3.4 Å, 5.48 Å, 3.39 Å, 3.28 Å, 6.18 Å, and 6.08 Å respectively. Apart from these Ala56, Pro357, Gly426 obtained an RMSF of 3.74 Å, 3.37 Å, 4.12 Å (Fig. 3a). Several of these residues lie in the GPGPG linker region of the vaccine construct. None of the above-mentioned residues was found to be involved in hydrogen bonding interaction with TLR-4 receptor as evident from the next section. The superimposition of 10 structural models of flexibility simulation of vaccine construct generated by CABS-flex has been shown in Fig. 3b.

### TLR-4 receptor and vaccine construct docking studies

**Fig. 4:**
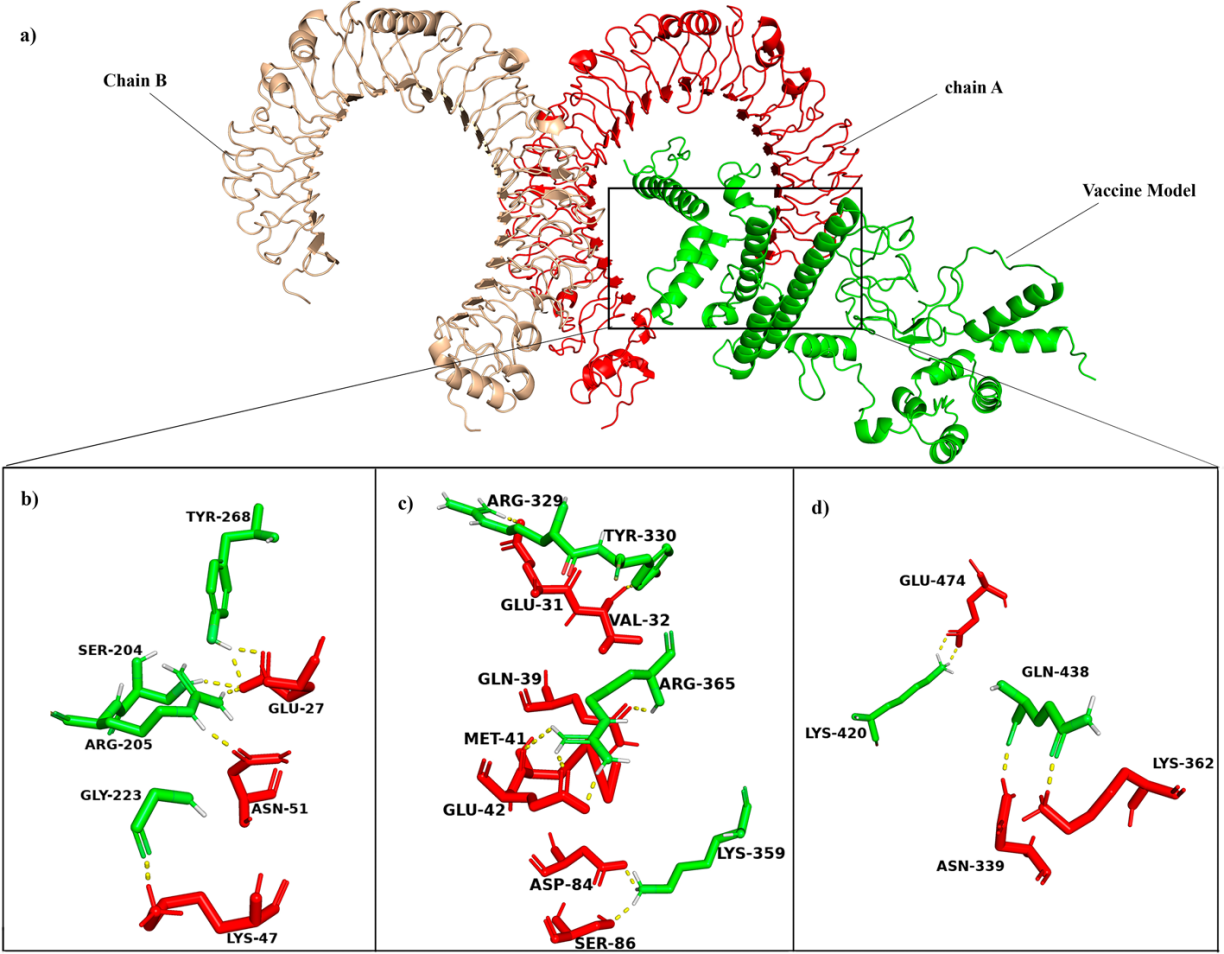
Receptor-Vaccine docking studies. **a** Vaccine model docked to TLR-4 receptor. Proposed model (Shown in green) found to interact with chain-A of TLR-4 (shown in red). **b**-**d** The interacting residues in the interface of TLR-4 and the vaccine model are visualised in Pymol and analyzed for presence of hydrogen bond (shown in yellow dotted line)

**Fig. 5:**
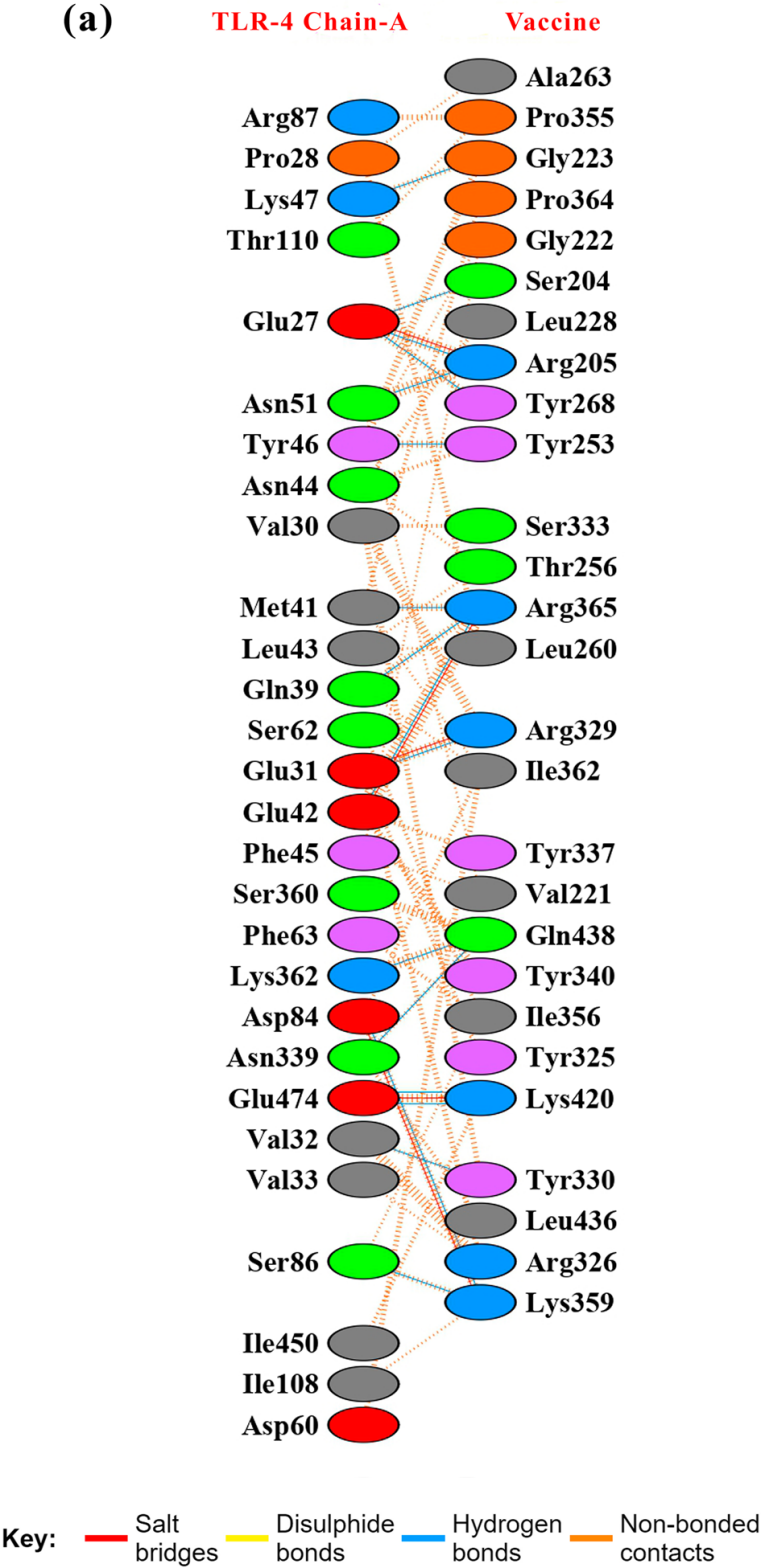
The interacting residues in the interface of docked complex. a List of Hydrogen bond interactions (shown in blue), salt bridges (shown in red), disulphide bonds (shown in yellow) and non-bonded contacts (in saffron) between the residues of vaccine construct and chain A of TLR-4 obtained from PDBsum

To analyze the binding affinity between TLR-4 receptor and vaccine model, docking studies were performed. Cluspro Server was used for docking TLR-4 receptor with the vaccine construct (Fig. 4a). Cluspro generated 30 models of different scores. From the top 10 models, we selected model 0 with largest cluster size of 61 members and possessing the lowest energy score of -1132.6 and carried for further studies. The hydrogen bond interactions between the TLR-4 and the vaccine were visualized in Pymol (Fig. 4b–d). PDBsum server further deciphered the interacting amino acid residues in the interface of a docked complex involved in Hydrogen bonds, salt bridge, and disulfide bond formations as well as non-bonded contacts (Fig. 5). 17 hydrogen bonds and 5 salt-bridges were formed in between TLR-4 and vaccine model as indicated by PDBsum results. Gibbs free energy or ΔG value was obtained from the PRODIGY server to further re-confirm the binding affinity of the vaccine towards the TLR-4 receptor. A negative Δ*G* value of − 13.3 kcal mol^−1^ and dissociation constant (k_d_) of 1.8E-10 obtained for our docked complex indicates that such interactions are energetically feasible.

### Prediction of conformational B-cell epitopes

A total of 7 discontinuous or conformational B-cell epitopes were predicted for the modeled vaccine ranging between scores of 0.574 to 0.804. A total of 223 amino acids were found to lie within these 7 epitopes with the size of the epitopes ranging from 3 to 101 amino acids. Table 6 shows the predicted conformational epitopes.

**Table 6:** Discontinuous *B*-cell Epitopes of final vaccine 3D model

Sl No	Residues	No of residues	Ellipro score
1	A:A375, A:I376, A:T377, A:V378, A:K379, A:K380, A:A381, A:G382, A:R383, A:L384, A:D385, A:N386, A:A387, A:T388, A:N389, A:K391, A:A392, A:Y394, A:P395, A:G396, A:M398, A:K399, A:A402, A:T403, A:D404, A:Y405, A:A406, A:A407, A:E408, A:A409, A:E410, A:R411, A:R412, A:A413, A:L414, A:S416, A:T417, A:W418, A:K419, A:K420, A:S421, A:R422, A:L423, A:P424, A:C425, A:G426, A:S427, A:L428, A:C429, A:C430, A:A431, A:P432, A:P433, A:L434, A:H435, A:L436, A:S437, A:Q438	58	0.804
2	A:M1, A:A2, A:K3, A:L4, A:S5, A:T6, A:D7, A:E8, A:L9, A:L10, A:D11, A:A12, A:F13, A:K14, A:E15, A:M16, A:T17, A:L21, A:D23, A:F24, A:V25, A:K26, A:K27, A:F28, A:E29, A:E30, A:T31, A:F32, A:V34, A:T35, A:A36, A:A37, A:A38, A:P39, A:V40, A:A41, A:V42, A:A50, A:G51, A:A52, A:A53, A:V54, A:E55, A:A56, A:A57, A:E58, A:A69, A:A70, A:G71, A:D72, A:K73, A:I75, A:G76, A:K79, A:V80, A:V81, A:R82, A:E83, A:I84, A:V85, A:S86, A:G87, A:L88, A:G89, A:L90, A:E92, A:P101, A:L104, A:K107, A:V108, A:A109, A:K110, A:E111, A:A112, A:A113, A:D114, A:E115, A:A116, A:K117, A:A118, A:K119, A:L120, A:E121, A:A122, A:A123, A:G124, A:A125, A:T126, A:V127, A:E131, A:A132, A:A133, A:A134, A:K135, A:E136, A:I137, A:K138, A:E139, A:A140, A:V141, A:L142	101	0.715
3	A:G231, A:P232, A:G233, A:P234, A:G235, A:A236, A:S237, A:S238, A:P239, A:F240, A:S241, A:S242, A:T243, A:S245, A:S246, A:S247, A:S248	17	0.709
4	A:A275, A:A276, A:Y277, A:L278, A:P279, A:M280, A:A281, A:A282, A:D283, A:T284, A:P285, A:A286, A:A288, A:Y289, A:F314, A:S315, A:A316, A:A317, A:G320, A:R321, A:F322	21	0.672
5	A:T344, A:L345, A:A346, A:A347, A:A348, A:Y349, A:S350, A:P351, A:W352, A:L353, A:S354, A:P355, A:I356, A:P357, A:H358, A:K359, A:I362	17	0.626
6	A:G151, A:P152, A:G153, A:P154, A:G155, A:M156	6	0.609
7	A:D304, A:D305, A:Y306	3	0.574

### In-silico immune-simulation studies

**Fig. 6:**
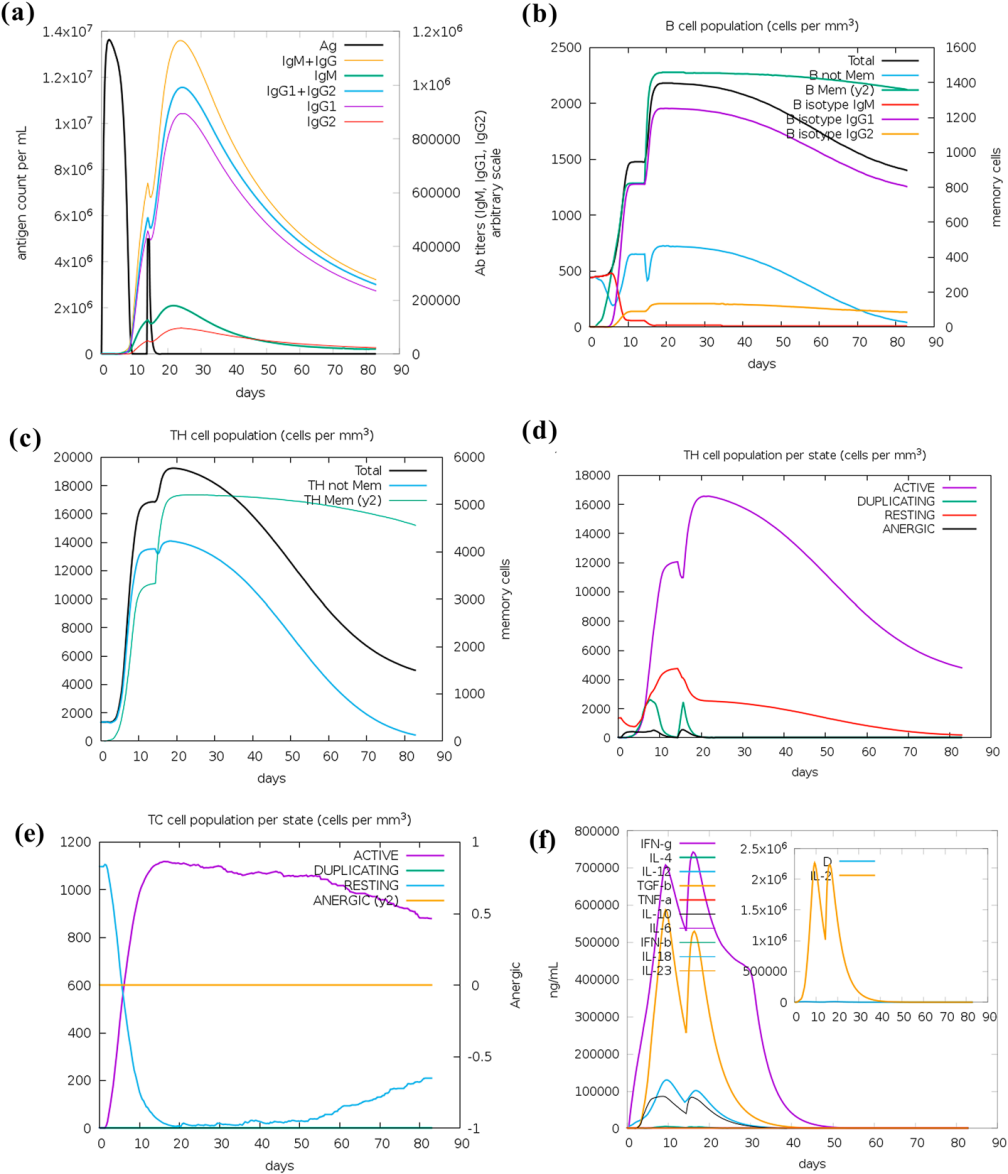
Immune simulation with vaccine construct. **a** Generation of antibodies after two doses of vaccine. The different isotypes and combinations of antibodies are shown in coloured peaks and antigen in black. **b** B-cell response after vaccine doses with coloured peaks denoting various subsets of B-cells. **c** Memory *T*-Helper cells and Total *T*-Helper cell proliferation curve. **d** Active and resting *T*-Helper cell population after vaccine injection. Resting *T*-cells are those which have not encountered the antigen and anergic cells signifies the *T*-cells which are tolerant to antigens **e** Active and resting Cytotoxic *T*-cell proliferation after vaccine dose. **f** The various cytokine profiles shown in coloured peaks with prominent up-regulation of pro-inflammatory IFN-*γ* (shown in violet). The insert graph shows simpson index D of IL-2 where D is measure of diversity

Immune-simulation was accomplished by utilizing C-immsim web server which can simulate both humoral and cell-mediated immune response *in-silico*. After application of the first dose, the antigen count reached around 1.3 × 10^7^/mL and dipped to count 0 on 10th day. A markedly high amount of IgG + IgM and slightly lower IgG1 + IgG2 population was noted after 10 days of the first vaccine dose as part of the primary immune response which was further amplified after the application of the second dose (Fig. 6a). IgM + IgG and IgG1 + IgG2 levels reached a peak value of around 1.1 × 10^6^ and 1 × 10^6^ on an arbitrary scale after 10 days of the second dose. The antigen count after the second dose was far less in contrast to the primary dose attaining a level of 5 × 10^6^/mL. Total B-cells count increased to 1500 cells/mm^3^ after 10 days of primary dose and reached a short plateau after that, which further increased attaining a peak of 2200 cell/mm^3^ after 5 days of the second dose and decreased gradually after that (Fig. 6b). The level of up-regulated memory B-cells lasted till 90 days which was the entire simulation period. B-cells of subtype IgG1 were predominantly high after 5–6 days of the primary shot. Apart from this, activation of cell-mediated immunity was accomplished as evident from the increased population of active CTL and HTL as well as memory T-Helper cells. A clear indication of proliferation of memory T-Helper cell has been shown in Fig. 6c. Active T-Helper cell population rose to 12,000 cell/mm^3^ after 12 days of primary dose and attained a peak after 5 days of secondary dose (Fig. 6d). The active cytotoxic T-lymphocytes attained a population higher than 1000 cells/mm^3^ after the first shot which remained stable till the 50th day (Fig. 6e). The number of resting cytotoxic T-cells population dropped vigorously after the first vaccine dose. Lastly, the level of pro-inflammatory cytokine IFN-*γ* was hugely amplified but in contrast to that, a very low level of anti-inflammatory cytokine IL-10 was noted (Fig. 6f).

### Molecular dynamics simulation and free energy calculations

**Fig. 7:**
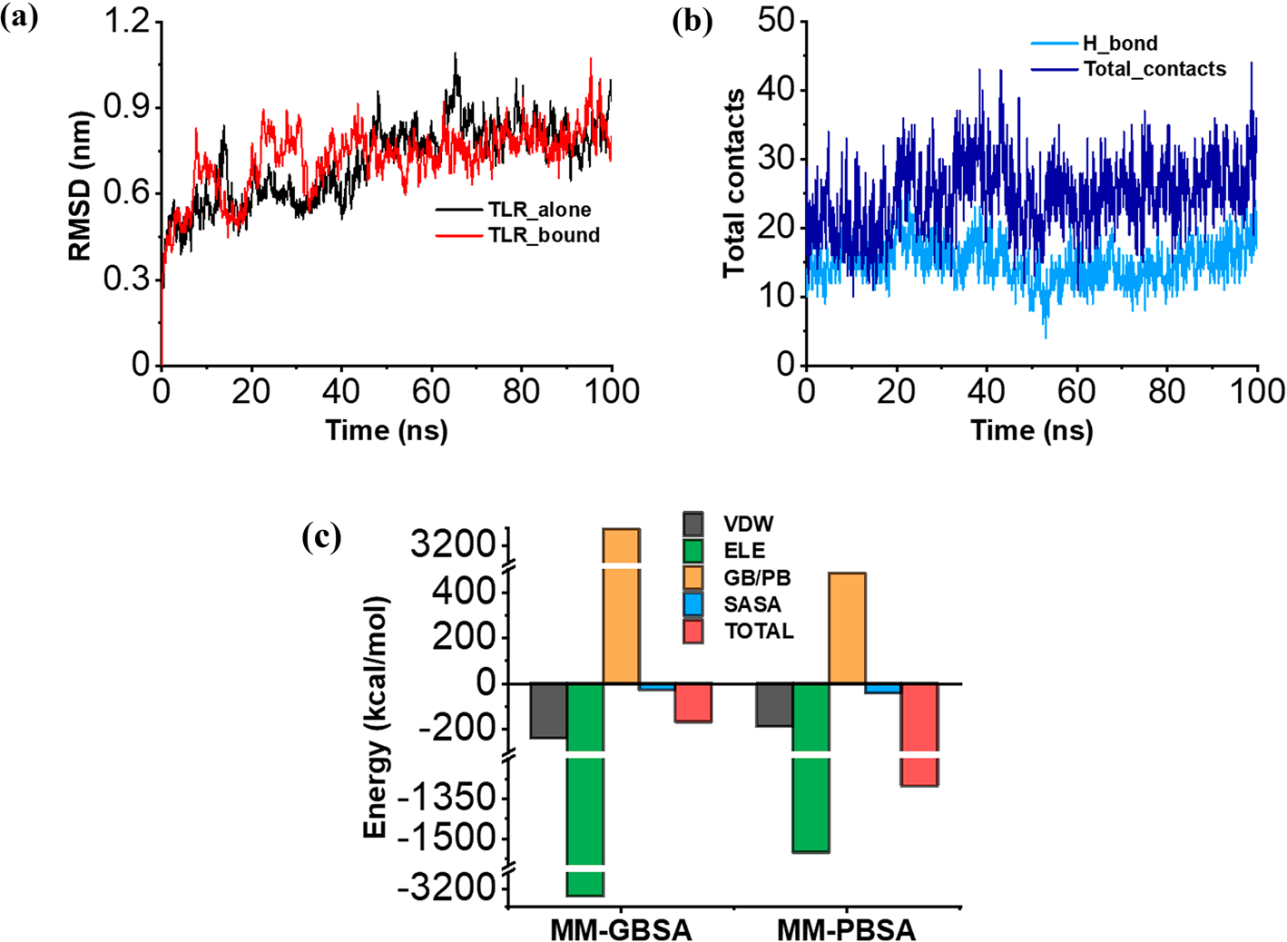
The stability and binding of TLR-4 with the vaccine accessed through MD simulations. **a** The RMSD of TLR-4 bound to the vaccine (Red) and TLR-4 alone (black) indicating improved structural stability upon binding. **b** The number of hydrogen bonds (sky blue) and the overall contacts (royal blue) between the TLR-4 and vaccine remains high and consistent during MD run. **c** The contributions of Van der Waal (black), Electrical (green), Poisson-Boltzmann (PB)/Generalized Born (GB) (Yellow) and SASA (blue) on the total binding energy (Red) of TLR-4 with the vaccine accessed by MM-GBSA and MM-PBSA approach

The global stability of the TLR-4-vaccine complex was studied through MD simulation. The root mean square deviations (RMSD), representing variations in the structures during the simulation period compared to the time zero state, are obtained. The complex stabilised earlier in the simulation trajectory compared to the TLR-4 alone and remained consistent, indicating enhanced stability (Fig. 7A). The stability is further assessed by root mean square fluctuations (RMSF) of the amino acid residues in the TLR-4 receptor. They also showed reduced fluctuations, indicating improved stability of the complex (Additional file 1: Fig. S2A). Insights from the local analysis suggested the formation of a large number of hydrogen bonds and total contact numbers between the vaccine and TLR-4, which remained consistent over the trajectory (Fig. 7B). Additionally, the distance between the vaccine and the TLR-4 receptor remains constant across the simulation time, indicating that the vaccine remains stable inside the binding pocket (Additional file 1: Fig. S2B). Further, the binding energy between the vaccine and TLR-4 receptor was calculated using the molecular mechanics Poisson-Boltzmann/Generalised Born surface area (MMPBSA, MMGBSA) approach. The free energy calculated is the cumulative effect of different energies involved in the binding process (electrical, van der Waal, solvent accessible surface area, and polar solvation energy). The polar solvation energy is calculated based on the Poisson-Boltzmann equation in the case of MMPBSA and the Generalised Born equation in the case of MMGBSA. The free energy analysis indicated a strong binding affinity of the vaccine towards the TLR-4 receptor with the binding energy of − 169.75 kcal/mol (MM-GBSA) and − 1304 kcal/mol (MMPBSA, Fig. 7C). The electrical and polar solvation energy played a significant role in the vaccine and TLR-4 binding (Fig. 7C). Conclusively, the MD simulations and free energy analysis indicated that the TLR-4 vaccine complex remains stable, and the vaccine has a high binding affinity toward the TLR-4 receptor.

### Codon optimization and In-silico cloning

**Fig. 8:**
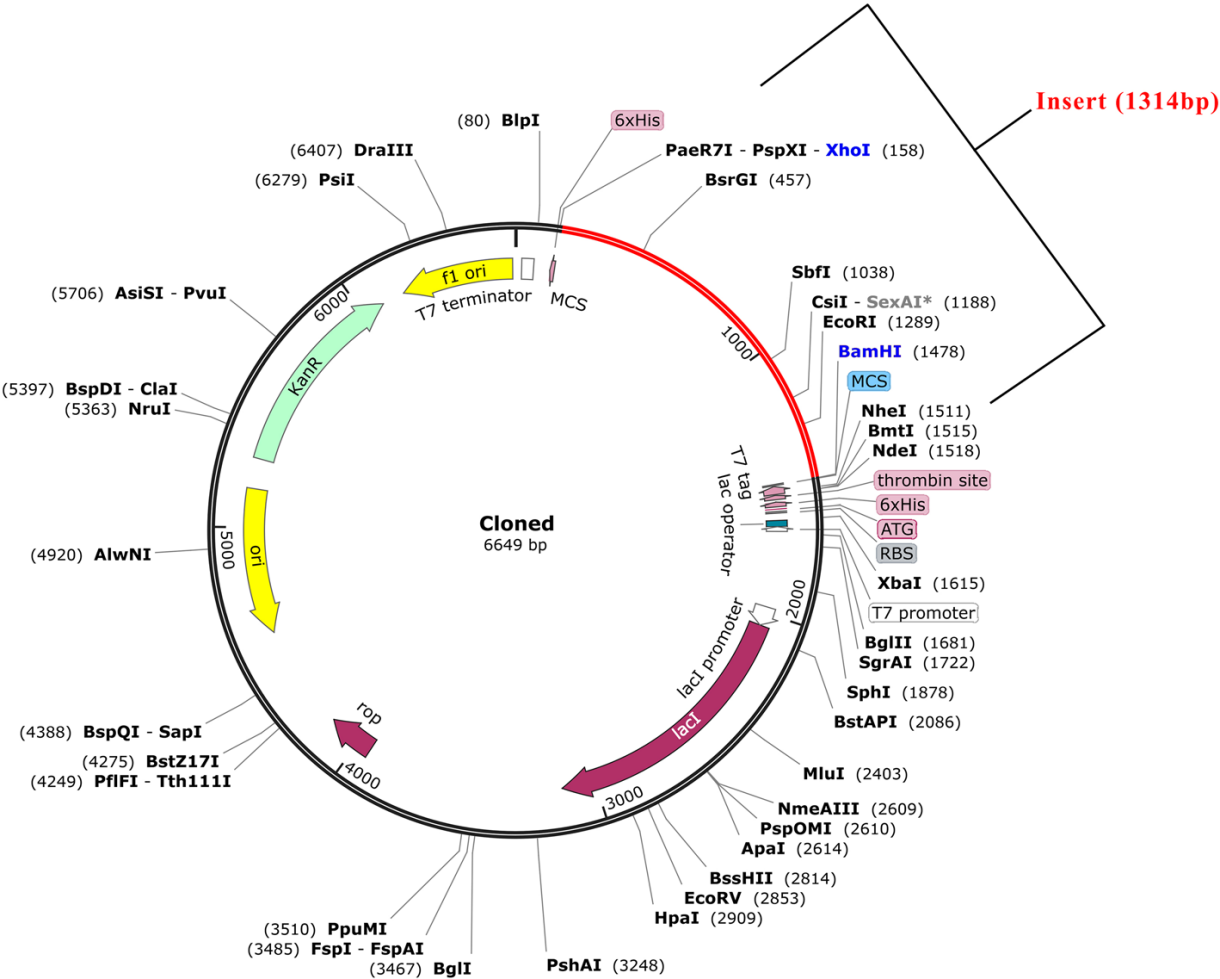
In-silico cloning of *Leishmania* vaccine construct into pET28A(+) vector. The red region denotes the 1314 bp cloned vaccine nucleic acid sequence. The restriction site labelled in blue are used for cloning

The final step is the cloning of the vaccine nucleic acid sequence into an expression vector. Java codon adaptation tool (JCAT) server was used for reverse translation of amino acid sequence and codon optimization against *E. coli* K12 strain. The output cDNA sequence scored Codon Adaptation Index (CAI) value 1 and GC% = 52%. A CAI value between 0.8 and 1 and GC% between 30 and 70% is ideal [41]. BamHI and XhoI sites were introduced at the 5’ and 3’ ends of the vaccine sequence for the generation of sticky ends after restriction digestion. Snapgene software was utilized to clone the vaccine construct into the pET28a(+) vector. The total obtained length of circular plasmid pET28a(+) along with the insert was found to be 6649 base pairs, while the insert size was 1314 base pairs (Fig. 8).

## Discussions

Leishmaniasis is a global health issue with no licensed vaccines for humans currently. Several chemotherapy and drugs which are available as treatment options suffer due to toxicity and resistance [42]. Vaccination is one the most efficient option available to affordably and rapidly control these diseases. Several methods of vaccine preparation like killed parasites, subunit vaccines, and DNA vaccines have been tested and have been found successful against *L. donovani.* But the conventional methods of vaccine preparation are time-consuming and can take up to a decade for development [43]. Immunoinformatics-based vaccine development can sometimes speed up the procedure by predicting the potential epitopes of a protein and the nature of the vaccines beforehand which is later employed for multi-epitope vaccine construction. These multi-epitope vaccines have certain advantages over the monovalent vaccines in terms of potency to cumulatively raise innate, humoral, and cellular immune responses [44].

In this study, it has been tried to construct a multi-epitope *Leishmania* vaccine, based upon *in-silico* immunoinformatics studies. Four *L. donovani* antigenic proteins sequences from NCBI were selected to map the HTL, CTL, and B-cell epitopes in them. HTLs are absolutely necessary for various infections due to their ability to release pro-inflammatory cytokines, inducing B cells for antibody secretion and class switching as well as activating macrophages. Belkaid et al., 2020 [45] reported that cytotoxic T-lymphocytes are important in low dose parasite infection and help in raising a secondary immune response. A reduction in splenic parasite load by 90% was observed by Polley et al., 2006 [46] on adoptive transfer of antigen-specific CTL during chronic *L. donovani*. Mixed reviews have been obtained regarding B-cells role in intracellular parasite infections like *Leishmania*. It has been shown by Mitchell et al., 1983 [47] that transfer of serum from healed mice does not confer protection in *Leishmania* but on the other hand, Woelbing et al., 2006 [48] reported that IgG antibodies in *L. major*-infected C57BL/6 mice up-regulated antigen uptake by dendritic cells.

All the shortlisted antigenic proteins, except ALSP, had a large number of antigenic HTL and CTL epitopes. Among these antigens, those having high affinity for host MHCs, IFN-γ inducing capability, and larger population coverage were chosen. CTL epitopes for allelic subtypes A1, A2, and A3 were not selected for ALSP due to their weak antigenicity. The B7 epitope of ALSP was selected which in spite of being antigenic showed less immunogenicity compared to other CTL epitopes. The 438 amino acid vaccine was constructed by joining the shortlisted 6 HTL, 9 CTL, and 4 B-cell epitopes of the four proteins with proper linkers along with an adjuvant as described before. The physicochemical parameters were analyzed by the ProtParam server, with a low instability index (< 40), confirming the construct to be sufficiently stable. Moreover, a higher GRAVY score indicated the hydrophobic nature of the construct, appropriately supported by the high aliphatic index, designating it as a safe, thermostable, alternative vaccine. AllerTOP server predicted the vaccine to be non-allergic.

The cumulative population coverage of the shortlisted T-cell epitopes was found to be 100% percent of the world population signifying the vaccine model can induce protective immunity against leishmania across the world. On evaluating the conservancy of the shortlisted epitopes it was found that all the protein epitopes except ALSP, were fairly conserved among the three *Leishmania donovani* strains BPK282A1, HU3, and CL-SL. Moderate to lower conservancy among other species suggested that the vaccine may not be effective against the *Leishmania major* and the *Leishmania braziliensis* infection. But as the vaccine is mainly designed to protect against visceral leishmaniasis caused by *L. donovani*, such observations may not be significant. The construct was 3D modeled using RaptorX, refined using GalaxyRefine and the output model was found to be valid as supported by PROCHECK Ramachandran plot data and the Z-Score obtained from ProSA. The refined model showed 89% residues in the most favored zone and only 0.3% in the disallowed zone in Ramachandran Plot analysis, signifying the model to be of high quality and is clearly better than unrefined RaptorX output. Further, the model was confirmed to be valid from the Z-Score value of – 6.37 which lies in the range of commonly found native proteins of similar size derived experimentally. The flexibility simulation graph of the vaccine model obtained from the output of CABSflex pointed out high fluctuations in RMSF values in various amino acids residues throughout the vaccine, especially in the region between 133–241 amino acids. These fluctuations illustrated that our model has high flexibility and validated its potential vaccine structure. The vaccine model and TLR-4 construct were docked to analyze the interactions and stability of such complexes. In-depth analysis and visualization of the docked complex showed 17 hydrogen bonds, and 5 salt-bridges between the vaccine model and the receptor indicating a high binding affinity between the two. The ΔG value of the docked complex obtained from the PRODIGY server is an important parameter because it denotes whether such interaction can actually take place. The negative ΔG value of -13.3 kcal mol^−1^ of the docked complex indicates the formation of the complex to be favorable. Additionally, the stability and the strength of binding between the vaccine and TLR-4 complex were analyzed through molecular dynamic simulations. The behaviour of the complex during a computationally derived dynamic environment provides a better outlook of the mechanics of interactions that play a role in the binding process. During the MD simulations the complex swiftly attained stability and showed high number of hydrogen bonds and overall contacts, which remained stable across the simulation period. Similar results were obtained during the free energy calculations, where the vaccine and TLR-4 showed high binding energy scores. The results obtained through MD simulations reinstates our docking results confirming the stability and high binding affinity of vaccine towards TLR-4 receptor.

The sole purpose of a successful vaccination is to induce both cell-mediated and humoral immunity efficiently to control the fatal infection [49, 50]. On *In-silico* testing of the capability of our vaccine construct to initiate an immune reaction it was observed that the target construct can efficiently raise a prominent T-cell and B-cell-mediated response. The population of CTL and active HTL increased after the first dose and further amplified after the second one, indicating efficient activation of cell-mediated immunity. A similar trend was observed for B-cells followed by elevated IgM and IgG levels after the initial dose. The vaccine construct does induce inflammation which was again confirmed by a higher level of pro-inflammatory IFN-γ and very less IL-10. The Simpson index was found to be insignificant, suggesting a diverse B and T-cell response. The increased number of memory B and T cells indicated that the vaccine can prime the immune system for future infections. The final step is the reverse translation and codon optimization of vaccine construct for high-level expression in *E. coli* system. The optimized cDNA sequence scored CAI of 1 and GC% = 52 which is appropriate for efficient expression of the proteins in the host system.

## Conclusion

Summarizing all the data it is evident that the proposed construct can be a promising multi-epitope vaccine model in near future. The vaccine is antigenic and has the potential to induce an immune response with production of IFN-γ. Structural analysis of the 3D model of construct reveals the structure to be legitimate and stable. The vaccine model has been hypothesised to display a good affinity towards the TLR-4 receptor upon binding, which can mediate a pro-inflammatory signalling pathway. We propose that the developed construct can be effective in humans all around the world (for the T-cells epitopes) exhibiting a population coverage of 100%. The current research further revealed that the epitopes are fairly conserved among the three selected strains of *L. donovani* and moderately conserved in *L. infantum,* indicating its wide applicability. The simulation studies clearly confirmed our vaccine construct as a good binder to the TLR- 4, implying its effectiveness. The novel observations have a futuristic approach to develop an anti-leishmanial chimeric vaccine candidate with predicted antigenicity and protective efficacy validated in in vitro and in vivo study set up.

## Methods

### Sequence retrieval and antigenicity analysis

The sequences of all the four proteins ATP-dependent Zinc Metallopeptidase (Accession no: XP_003863163), Histidine Secretory Acid Phosphatase (XP_003865788), Rhomboid like protein (XP_003857936.1), and ALSP (XP_003864424) were retrieved from NCBI. 50S ribosomal protein L7/L12 (130 amino acid) sequence was collected from Uniprot (P9WHE3). Vaxijen Server (http://www.ddg-pharmfac.net/vaxijen) was employed for predicting the antigenicity of each of the proteins [51]. A default threshold value of 0.5 for parasites was used throughout.

### HTL Epitope prediction

Helper T-lymphocytes play an important role in defence against invading pathogens by activation of macrophages to destroy ingested pathogen as well as aiding cytotoxic T-cells to eliminate target cells and maturation of B cells [52, 53]. Identification of Helper T-lymphocytes epitope or MHC-II binding epitope was accomplished using IEDB (http://tools.iedb.org/mhcii) and NETMHCII-2.3 server (http://www.cbs.dtu.dk/services/NetMHCII). At first, the sequence data of each four proteins were uploaded to the IEDB server, the consensus 2.22 method was selected and the reference set of 27 alleles was checked.

15-mer epitopes having percentile rank ≤ 1 and SMM align IC50 ≤ 50 nM were chosen for further analysis. An IC50 of ≤ 50 nM signifies higher affinity whereas a value 50 nM < IC_50_ ≤ 1000 nM shows intermediate affinity and values 1000 nM < IC_50_ ≤ 5000 nM shows low affinity [54, 55]. The shortlisted epitopes were further filtered based on the antigenicity using Vaxijen server and a threshold of 0.5 for the parasite was selected. The antigenic epitopes were screened for the IFN-*γ* and IL-10 induction using IFN-Pred (https://webs.iiitd.edu.in/raghava/ifnepitope) and IL-10 Pred (https://webs.iiitd.edu.in/raghava/il10pred) [56, 57]. IL-10 has been reported to reduce inflammation thereby aiding parasite replication inside the macrophages [58]. Naturally, inhibiting the IL-10 hinders parasite replication [59]. Pro-inflammatory cytokine IFN-γ has been found to promote parasite clearance in visceral leishmaniasis [60, 61]. The epitopes found to be IFN-γ inducing and IL-10 non-inducing were again checked for their binding affinity (both strong binding and weak binding) using NETMHCII 2.3 server against a set of 27 alleles that cover majority of the world population. NETMHCII server was created using an extended data set consisting of data regarding quantitative MHC-peptide binding affinity obtained from IEDB covering HLA-DQ, DR, DP, and mouse H-2 alleles [62]. The default threshold of 2% for strong binders and 10% for weak binders was maintained. The epitopes found to bind a wider number of alleles among the selected allele set in the NETMHCII 2.3 server were selected for vaccine construction. The set of alleles selected in IEDB and NETMHCII 2.3 is given in Additional file 1: Supplementary information 1A.

### CTL Epitope prediction

Cytotoxic T-lymphocytes confer protection to visceral leishmaniasis and elevated level of CD8 + T-lymphocytes along with Granzyme B have been found in healed visceral leishmaniasis subjects [63, 64]. NETCTL 1.2 (https://services.healthtech.dtu.dk/service.php?NetCTL-1.2) server was used to predict potential 9-mer CTL epitopes. Each of the four protein fasta was uploaded and searched for CTL epitopes against A1, A2, A3, and B7 allele subtypes. A default threshold score of 0.75 was maintained throughout. NETCTL server integrates epitope prediction based upon binding of MHC-I, Proteasomal cleavage, and transport efficiency of TAP and produces a combined score based on all these factors [65]. The predicted epitopes having a combined score of more than 0.75 were checked for antigenicity using Vaxijen and immunogenicity using IEDB class I immunogenicity server (http://tools.iedb.org/immunogenicity). The high-scoring epitopes were shortlisted and further checked for their binding affinity to a wider number of alleles using NETMHC 4.0 server maintaining the default parameters. NETMHC 4.0 uses an Artificial Neural Network based gapped sequence alignment to predict MHC class-I peptides [66]. Epitopes found to have a high binding affinity towards a wider number of alleles, as evident from NETMHC 4.0, were selected. The set of alleles selected for our study using the NETMHC 4.0 server is given in Additional file 1: information 1B.

### Linear B-cells epitope prediction

B-cells regulate the humoral immunity and produce antibodies which is an important aspect of every vaccine design. ABCpred (https://webs.iiitd.edu.in/raghava/abcpred) server which is built upon an artificial neural network was used to predict the linear B-cell epitopes [67]. 18-mer antigenic epitopes returned with the highest score for each protein were included in the final vaccine construct. The antigenicity of the epitopes was predicted using Vaxijen.

### Construction of chimeric multi-epitope vaccine.

To construct an effective vaccine all the shortlisted HTL, CTL, and B-cell epitopes were arranged linearly and fused to form a chimera. Certain linkers like GPGPG to join HTL epitopes, AAY to join CTL epitopes, and KK to join B-cell epitopes were used as reported previously [26, 68]. These linkers play a vital role to improve protein stability by introducing flexibility, protein folding, and separation of multiple domains [69]. A TLR-4 agonist 50S ribosomal protein L7/L12 which enhances vaccine interaction with TLR-4 receptor was integrated to N-terminal of the vaccine construct using an EAAAK linker [70]. These adjuvants behaving as agonists play a critical role in enhancing vaccine efficacy [71].

### Physicochemical characterization of vaccine and glycosylation analysis

The Expasy Protparam tool (https://web.expasy.org/protparam) was used for physicochemical characterization of vaccine construct. Several parameters like Theoretical pI, Molecular Weight, Aliphatic Index, In-vitro and In-vivo half-life, GRAVY, and instability index were assessed. The instability index of the protein indicates the protein stability. A value of such for the entered protein sequence < 40 suggests the protein to be stable. The in-vivo half-life of the protein sequence is evaluated using "N-end rule" which is related to amino-acid residues at the N-terminal region [72]. Aliphatic Index is the measure of thermal stability of the protein and a higher value proposes more stability [70, 73]. The GRAVY score indicates the hydrophobicity of the protein. It is obtained by dividing the total hydropathy of all amino acid by the number of amino acids [74]. A positive GRAVY score specifies a hydrophobic nature of the protein, on the other hand, a negative score indicates hydrophilic characteristics [75]. Glycosylation of the protein is a post-translational modification often involved in efficient protein folding and transportation. In order to predict the presence of overlapping glycosylation sites of the vaccine epitopes with target proteins, NetNglyc 1.0 (https://services.healthtech.dtu.dk/service.php?NetNGlyc-1.0) was deployed [76].

### Evaluation of epitope conservancy and population study

Epitope conservancy among the various species and strains of a pathogen are prerequisite for designing of a broad spectrum chimeric vaccine. All the CTL, HTL and B-cell epitopes shortlisted were applied to this pipeline for conservancy analysis. Three different strains of *Leishmania donovani* namely BPK282A1, CL-SL, and HU3 were considered for epitope conservancy analysis. Besides this *L. infantum* strain (JPCM5), *L. major* (friedlin) and *L*. *braziliensis* (MHOM/BR/75/M2903) were included in the study. IEDB epitope conservancy tool (http://tools.iedb.org/conservancy/) [77] was employed at a threshold value of ≥ 100% and the sequence homology of selected epitopes among various species and strains were obtained. MHC molecules are extremely polymorphic in nature and vary extensively amongst people residing in various geographical locations. To study the cumulative population coverage of the T-cell epitopes (Both MHC I AND II) IEDB population coverage tool (http://tools.iedb.org/population/) was used [78]. The selected epitopes and corresponding binding alleles were submitted to the server and world population coverage was assessed.

### Allergenicity and antigenicity evaluation of the chimeric construct

The Allergenicity of the vaccine was evaluated using the AllerTOP server (https://www.ddg-pharmfac.net/AllerTOP). The AllerTOP functions based on auto cross-covariance (ACC) transformation of the protein sequences into uniform equal-length vectors [79]. Vaxijen server was used to evaluate antigenicity.

### Secondary and tertiary structure prediction of vaccine construct

PSIPRED web server (http://bioinf.cs.ucl.ac.uk/psipred) was employed to generate the secondary structure of the vaccine construct. It is a highly accurate prediction method and incorporates two feed-forward neural networks which analyze output obtained from PSI-BLAST (Position-specific integrated-BLAST) [80]. Modeling of the 3D structure of the protein was accomplished by RaptorX web server available at https://raptorx.uchicago.edu. RaptorX utilizes a distance-based protein folding powered by deep learning [81]. It can prepare a 3D model of proteins lacking close homologs in PDB. This server has ranked first in contact prediction in CASP 12 and 13.

### Tertiary structure refinement and validation

The 3D model of the vaccine construct as obtained from RaptorX was exported to GalaxyRefine server (http://galaxy.seoklab.org/cgi-bin/submit.cgi?type=REFINE) for further refinement. This server rebuilds the side chain and carries out side-chain repacking and overall structural relaxation by applying molecular dynamics simulation [82]. For analysing the statistics of non-bond interaction between different atoms ERRAT web tool was used. Ramachandran Plot was generated using PROCHECK to further judge the quality of the predicted 3D model of the vaccine. The scores obtained from the Ramachandran plot signify the size of energetically favored regions and getting a score higher than 85% can be labeled as acceptable [83. Both tools are available under SAVES version 6 (https://saves.mbi.ucla.edu). Lastly, ProSA web tool was utilized to generate the Z-score of the modeled construct which was used to judge overall quality. The output of ProSA is given in form of a graph with a Z-score (y-axis) of structures of native proteins deciphered by NMR and X-ray crystallography against the residues (x-axis) [84]. CABS-flex (http://212.87.3.12/CABSflex2/index) server was used to analyze the flexibility of vaccine 3D model. It is a server utilized for fast modeling of protein structure flexibility [85]. Within nanosecond time, CABS-flex webserver shows Root Mean Square Fluctuations (RMSF) simulation of all amino acid residues present in a protein [49]. The *RMSF* is an estimate of the displacement of a group, or particular atoms, with respect to the reference structure, averaged over the number of atoms [86]. We selected 50 cycles and a temperature of 1.4° for simulation.

### Prediction of conformational B-cell epitopes

Discontinuous B-cell epitopes are formed by residues that are located far apart in protein sequence but are brought close to each other in spatial proximity by protein folding [87]. Ellipro (https://tools.iedb.org/ellipro) antibody epitope prediction server uses the 3D model of the protein to predict probable discontinuous or conformational B-cell epitopes [88]. ElliPro uses three algorithms which are based upon values of protrusion index (PI) for estimating the shape of the protein as an ellipsoid, measure the pI of the residues, and adjacent cluster residues [89]. The vaccine model in PDB format was uploaded and all default parameters were kept for epitope prediction.

### Molecular docking of TLR-4 receptor and vaccine construct

TLR-4 expression increases in leishmaniasis, and the receptor is involved in development of the infection [90]. It has been reported previously that TLR-4 increases expression of inducible nitric oxide synthase during *Leishmania major* infection [91]. Besides these, triggering of TLR-4 receptor do improve the outcome of chemotherapy and helps in developing resistance against parasite [92]. Protein–protein docking of the chimeric vaccine constructs and TLR-4 molecule (PDB ID: 4G8A) was carried out to analyze the affinity and interactions in the complex. ClusPro web server (https://cluspro.bu.edu/) was utilized for molecular docking studies. ClusPro uses three computational steps (i) PIPER based rigid body docking (ii) clustering of 1000 lowest energy docked structures through pairwise IRMSD as the distance measure (iii) Complex structures refinement which are located at cluster centres by minimization of their energy [93]. The binding free energy (ΔG in kcal mol − 1) of the docked complex from cluspro was obtained from the PRODIGY webserver (https://wenmr.science.uu.nl/prodigy/). The equation ΔG = RTln(K_d_) was used to calculate the dissociation constant K_d_, where R in the equation denotes the ideal gas constant (kcal K − 1 mol − 1), T the temperature (K) [94].

### In-silico immune response simulation

C-immSim (https://kraken.iac.rm.cnr.it/C-IMMSIM) is an online immune response simulation server that can predict both cell-mediated and humoral immune response profile in mammals after vaccination [95]. C-ImmSim is an agent-based model, which uses a matrix-based strategy for epitope forecasting and techniques based on machine learning to forecast immune interactions [96]. It simulates three anatomical components, the bones, where myeloid cells are produced from stimulated hematopoietic stem cells, the lymphatic organs, and the thymus where selection of naive T-cells takes place to prevent autoimmunity [97]. We simulated the injection of two doses of vaccines at an interval of 14 days. Therefore 10,000 number of antigens at time steps 1 and 42 were injected where each time step stands for 8 h of real life. Simulation steps were adjusted to 250. Default parameters were maintained for all other random seed, host alleles, and simulation volume.

### Molecular dynamic simulations

The molecular dynamic simulations of TLR-4 alone and TLR-4 vaccine complex were performed in the GROMACS version 2018 using the GROMOS 54a7 force field. The proteins were kept in a cubical box 10  Å equidistant from all sides and solvated using the SPC water model. The system was neutralised by adding appropriate charges, and energy minimisation was performed using the steepest descent method for 5000 steps. The system was further fed to NPT, NVT ensemble for temperature and pressure equilibration of 100 ps. The system was maintained at a constant temperature of 310 K using modified Berendsen coupling. For pressure coupling, Parrinello-Rahman barostat was applied with compressibility set to 4.5 × 10^–5^ bar^−1^. Long-range interactions were calculated using the Particle Mesh Ewald (PME) method, and velocity was generated using a leapfrog integer at a time step of 5 fs. Finally, the simulations were carried out for 100 ns, with coordinates saved every 10 ps [98–100]. The trajectory analysis was performed using the standard GROMACS module, and data analysis was done in origin pro software, version 2020b (OriginLab Corporation, Northampton, MA, USA).

### Free energy calculations

The free energy for the TLR-4 and vaccine interaction was calculated using MM-PBSA and MM-GBSA approaches (molecular mechanics-based Poisson Boltzmann/Generalised Born). These molecular mechanics techniques consider the contributions of bonded, van der Waals, electrostatic, polar, and non-polar components. The polar components in the case of MM-PBSA are calculated using the Poisson Boltzmann equation; here, the MD trajectory is utilised, and interactions are calculated accordingly [101]. In the case of MM-GBSA, the polar solvation component is calculated using the Generalised Born equation using the HawkDock server [102].

### Codon adaptation and in-silico cloning

The final step of vaccine designing is devising methods to express the protein in host organism efficiently. Here *in-silico* cloning was done to foresee the nature of protein expression. The amino acid sequence of the vaccine was uploaded to JCAT (http://www.jcat.de/) [103] and codon-optimized nucleic acid sequence was obtained against *Escherichia coli* K12 as the host organism. The cDNA sequence obtained for our vaccine model from the server was applied to NEBcutter to analyze the restriction enzyme cut sites available in the sequence. BamHI and XhoI sites were attached to 5’ and 3’ ends of the nucleic acid sequence and Snapgene software was used to restriction digest and clone the sequence into pET28(+) vector.

## Supplementary Information

**Additional file 1.** The Supplementary information includes MHC-I and MHC-II alleles used in this study. Supplementary figure 1 shows the world population coverage analysis. Supplementary figure 2 contains Molecular Dynamics Simulation additional files.

## Data Availability

The sequences of all four proteins ATP-dependent Zinc Metallopeptidase (Accession no: XP_003863163), Histidine Secretory Acid Phosphatase (XP_003865788), Rhomboid like protein (XP_003857936.1), and ALSP (XP_003864424) are available and retrieved from NCBI (https://www.ncbi.nlm.nih.gov/). The sequence of 50S ribosomal protein L7/L12 is downloaded from Uniprot (https://www.uniprot.org/). Pymol software has been downloaded from https://pymol.org/2/ and used under trial license. Snapgene was downloaded from https://www.snapgene.com/ and used under trial license.

## Abbreviations

ALSP: Amastin-like surface protein
CAI: Codon adaptation index
CTL: Cytotoxic T-lymphocytes
GBD: Global burden of disease study
GRAVY: Grand average of hydropathicity
HTL: Helper T-lymphocytes
IEDB: Immune epitope database
JCAT: Java codon adaptation tool
MD: Molecular dynamics
MHC: Major histocompatibility complex
NMA: Normal model analysis
PDB: Protein Data Bank
RMSD: Root mean square deviation
RMSF: Root mean square fluctuation
TLR-4: Toll like receptor-4
VL: Visceral Leishmaniasis
