# Supplementary material for: In-silico design of an immunoinformatics based multi-epitope vaccine against Leishmania donovani
Source: BMC Bioinformatics. 2022 Aug 5;23:319. doi: 10.1186/s12859-022-04816-6 (PMC9354309; doi:10.1186/s12859-022-04816-6)
Supplement: Supplementary file 1 — Additional file 1. The Supplementary information includes MHC-I and MHC-II alleles used in this study. Supplementary figure 1 shows the world population coverage analysis. Supplementary figure 2 contains Molecular Dynamics Simulation additional files. [file 12859_2022_4816_MOESM1_ESM.docx]

**SUPPLEMENTARY INFORMATION**

**1A) MHC Class-II alleles selected for analysis in IEDB MHCII AND NETMHCII 2.3 server**

DRB1*0101, DRB1*0301, DRB1*0401, DRB1*0405, DRB1*0701, DRB1*0901, DRB1*1101, DRB1*1201, DRB1*1302, DRB1*1501, DRB3*0101, DRB3*0202, DRB4*0101, DRB5*0101, DRB1*0802, HLA-DQA1*0501-DQB1*0201, HLA-DQA1*0501-DQB1*0301, HLA-DQA1*0301-DQB1*0302, HLA-DQA1*0401-DQB1*0402, HLA-DQA1*0101-DQB1*0501, HLA-DQA1*0102-DQB1*0602, HLA-DPA1*0201-DPB1*0101, HLA-DPA1*0103-DPB1*0201, HLA-DPA1*0103-DPB1*0401, HLA-DPA1*0301-DPB1*0402, HLA-DPA1*0201-DPB1*0501, HLA-DPA1*0201-DPB1*1401

**1B) MHC Class-I alleles selected for analysis in NETMHC 4.0 server**

HLA*A0101, HLA*A0201, HLA*A0202, HLA*A0203, HLA*A0205, HLA*A0206, HLA*A0207, HLA*A0211, HLA*A0212, HLA*A0216, HLA*A0217, HLA*A0219, HLA*A0250, HLA*A0301, HLA*B0702, HLA*B5801, HLA*B5802, HLA*B3501, HLA*B3503, HLA*B2705, HLA*B2720, HLA*B3901, HLA*B1501

**
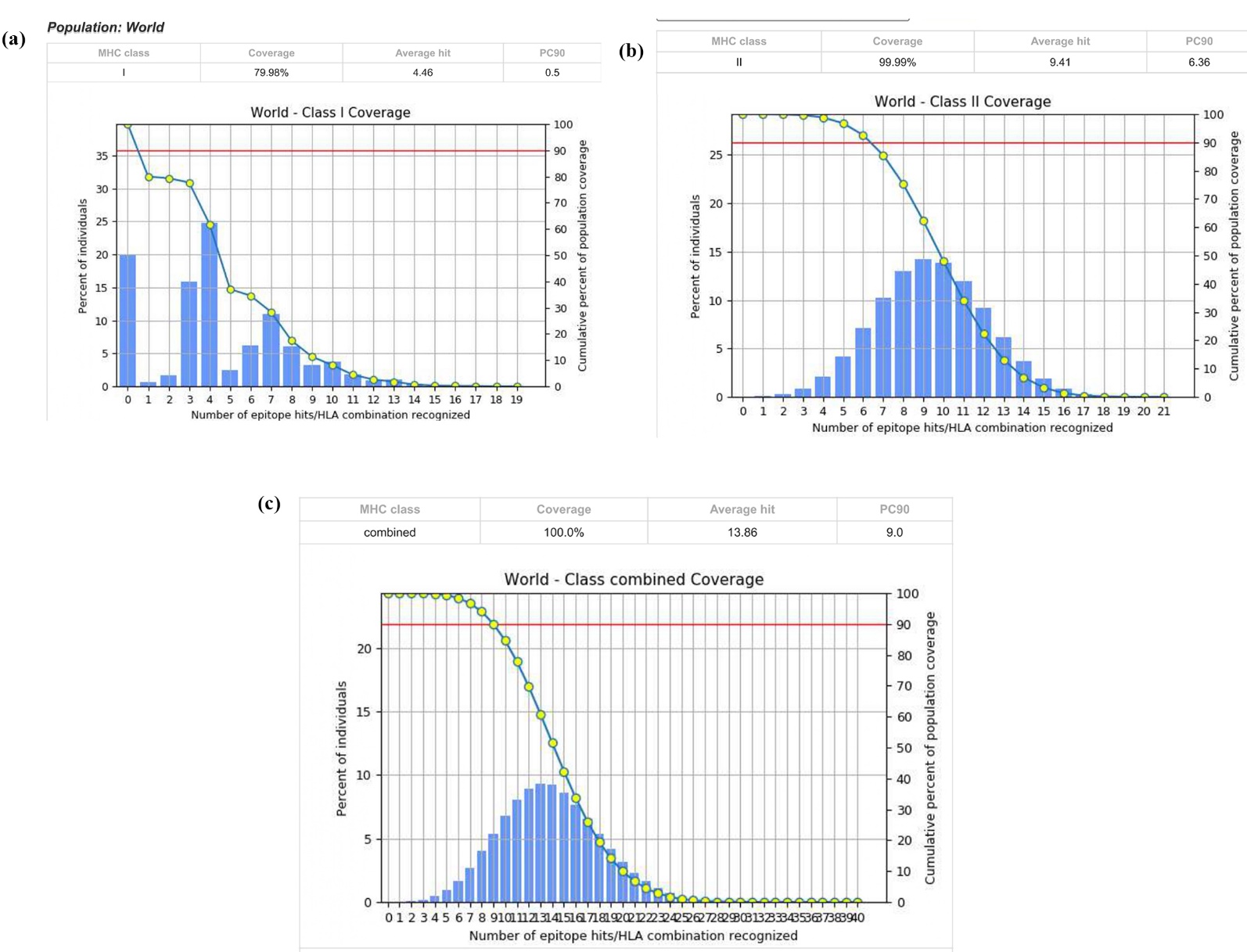
SUPPLEMENTARY FIGURES**

**Supplementary Figure 1:** Population coverage analysis**-** (a) MHC-I epitope coverage (b) MHC-II epitope coverage (C) combined MHC-I and II binding epitopes coverage.

**
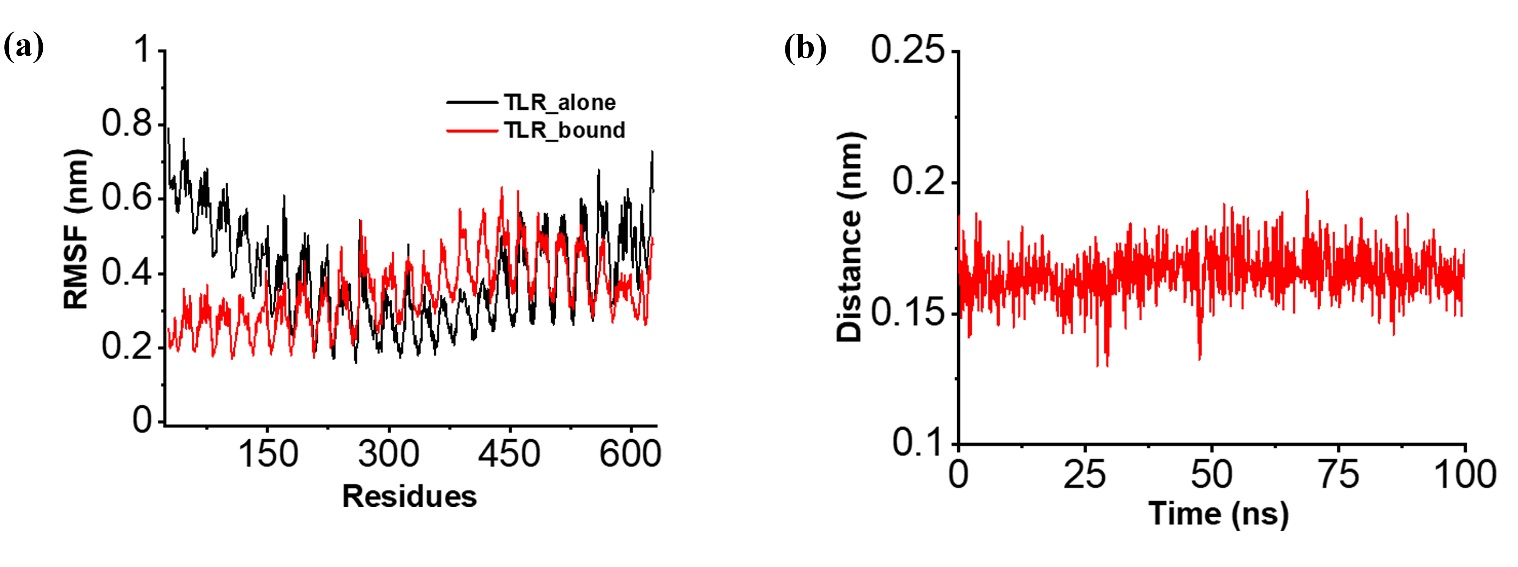
**

**Supplementary Figure 2:** The stability and binding of TLR-4 with the vaccine accessed through MD simulations. a) The root mean square fluctuations (RMSF) of TLR-4 bound to the vaccine (Red) and TLR-4 alone (black) indicating improved residue stability upon binding during the course of simulations. b) The distance between the TLR-4 and vaccine remains consistent during the simulation period.
